# The Pathology and Treatment of Epidemic Cholera

**Published:** 1866-04

**Authors:** N. S. Davis

**Affiliations:** Prof. Practical and Clinical Medicine, Chicago Medical College


### CHICAGO MEDICAL EXAMINER.

N. S. DAVIS, M.D., EDITOR.

VOL. VII. APRIL, 1866. NO. 4.

### Original Contributions.

### ARTICLE XIII.

### THE PATHOLOGY AND TREATMENT OF EPIDEMIC CHOLERA.

BY N. S. DAVIS, M.D., Prof. Practical and Clinical Medicine, Chicago Medical College.

In compliance with the wishes of some professional friends, I have written the substance of the remarks made by me in opening the discussion on the pathology and treatment of epidemic cholera, in the Chicago Medical Society, at a recent meeting. They were as follows:—

Owing to the very brief time allowed to each member of the Seciety in our discussions, I shall, on this occasion, make no remarks in regard to the causes of cholera, but simply assume that an efficient cause does exist, whether in the form of a specific poison, or in some peculiar combination of atmospheric conditions. Neither shall I spend any time in rehearsing the various theories that have been proposed, in relation to the nature of the disease; but, having had ample opportunities to study its phenomena and results, during five epidemics, I shall simply give the conclusions at which I had arrived.

In studying the phenomena or symptoms of cholera closely, from its initial disturbances to the complete collapse and death, we find, throughout, unmistakable evidence of a diminution of vital affinity, an impairment of sensibility in the excito-vascular nervous system, and increased susceptibility of the mucous membrane of the digestive organs. By vital affinity I mean that property inherent in all living organized tissues, which causes those atomic changes by which nutrition, disintegration, and secretion are effected and caloric evolved. This property not only regulates or determines the selection and appropriation of new organic atoms or cells constituting nutrition; the rejection of old atoms constituting disintegration; and the selection and combination of atoms or elements by the secreting cells, constituting secretion; but in the regulation of these functions it necessarily regulates the evolution of caloric and the *tonicity* of the tissues. It is independent of all mere nervous function or influence, and is capable of being increased, diminished, or perverted by the action of exterior influences, both healthful and morbific.

By the *excito-vascular* nervous system, is meant that part of the ganglionic or organic nervous system which follows the bloodvessels and supplies their coats with nerve influence. Some of these filaments are sensitive and are distributed to the interior lining of the capillary vessels, while others are motor and supply the muscular fibres entering into the composition of the capillary walls, and they bear the same functional relation to each other as the cerebro-spinal nerves of sensation and motion do. Thus, each impulse of blood from the heart into the capillaries makes an impression on the sensitive branches of the excito-vascular nerves and calls forth a reflex influence upon the muscular fibres of the capillary vessels, inducing sufficient contraction to make these minute vessels active instead of passive agents in carrying on the circulation. The word capillary is here used in its ordinary sense and includes both the arterioles and the *true* capillaries of Kolliker and Flint. As the atomic and cell changes which constitute nutrition, disintegration, secretion, and calorification depend directly upon the coöperation of a proper degree of vital affinity with a regular and healthful capillary circulation, whatever is capable of impairing the former or disturbing the latter will necessarily disturb, more or less, all the functions named. In my estimation, the cause or causes of cholera directly produce a decided diminution of vital affinity and a diminished sensibility of the arterioles or systemic arterial capillaries throughout the whole system, while the susceptibility or irritability of the mucous membranes is increased. This gives rise, first, to general diminution of tonicity, indicated by lassitude and muscular weakness; to feebleness of capillary circulation, indicated by paleness and coolness of skin, softness of pulse, and diminished glandular secretion; and to unnatural disturbance of the alimentary canal, in which the increased excitability and impaired tonicity leads to ready serous effusion and consequently liquid evacuations from the bowels. These constitute the essential phenomena of cholerine or the forming stage of active cholera. The pathological conditions, thus started, increase with a rapidity proportionate to the intensity of the action of the causes and the previous condition of the patient. In ordinary epidemic cases, a few hours is often sufficient to bring the relation between the vital affinity of the tissues and the movement of the blood in the capillaries to such a condition that all the nutritive and natural secretory action is stopped, the pulse is rapidly sinking, and all the tissues becoming cold, while the serous effusion from the mucous surfaces and skin is rapidly exhausting the water and salts of the blood, and so disturbing the circulation in the cerebro-spinal nervous centres as to cause the induction of irregular muscular spasms or cramps in any or all of the voluntary muscles. This constitutes the active stage of severe cholera.

During this stage, another pathological element comes up for consideration, namely, the rapid change in the fluidity of the blood, from the loss of the water and salts. This change adds still more to the embarrassment of capillary circulation and hastens the period of collapse, when the pulse ceases in the ex tremities, the tissues become shrunken and cold, the skin corru gated and bluish, the sphincters relaxed, and the mind torpid or inactive. It is in this stage that a large majority of the deaths take place; though when the cause or causes of an epidemic are acting in their highest degree of intensitv vital affinity and capillary sensibility may be so directly and fully suspended that all organic changes cease almost immediately, the patient becomes rapidly cold, blue or leaden color, pulseless, and dies with but little evacuations either by vomiting or purging.

With these views, in regard to the pathology of cholera, we have no difficulty in deducing certain rational indications for treatment. These indications are, to adopt such measures as will increase vital affinity and especially increase the sensibility and tonicity of the systemic capillaries; to allay the morbid irritability of the mucous membranes; to retard or suppress the discharge of the serum and salts of the blood through the mucous membranes and skin; and, at the proper stage of the disease, to supply, as directly and rapidly as possible, the materials lost from the blood in the excessive discharges. If the first two indications named could be fully met in the early stage of the disease, it would render the two latter unnecessary. But, unfortunately, so rapid is the progress of many cases of cholera, that they are not brought under the care of the physician until the first stage is already past, and the conditions on which all the above indications are founded are fully developed.

In the limited time allowed in this discussion, I cannot enter upon a general consideration of the various means or remedies that may be more or less useful in the accomplishment of the objects named, but will simply indicate those I deem most efficient:—

For increasing vital affinity and the sensibility of the systemic copillaries, and, thereby, sustaining capillary circulation, secretion, organic changes, and evolution of caloric I should have great confidence in the inhalation of oxygen gas as the most direct and efficient natural stimulant or excitor with which we are acquainted, if it could be at the command of the practitioner and its administration properly regulated. As a substitute, I would suggest that a fair trial be made with the nitrous-oxide gas.

Aside from these two gases, the well-known properties of strychnia in increasing nerve sensibility and muscular tonicity suggest it as a remedy of value; and there is on record much reliable clinical evidence in its favor.

To allay the morbid irritability of the mucous surfaces, the preparations of opium are almost universally acknowledged to be most efficient; and, when combined with astringents and alteratives, they also afford the most efficient known means for retarding or arresting the serous effusions from the mucous lining of the stomach and bowels.

My own practice, when called to a case of cholera in the early part of the active stage, when the rapid vomiting, purging, and perhaps cramps have just fairly begun, is to direct the immediate application of strong mustard sinapisms over the epigastrium and nearly the whole length of the spine; keep the patient in a horizontal position, with dry warmth to the extremities; and give internally, every half-hour, the twentieth of a grain of strychnia with 10 drops of oil of turpentine, in the form of an emulsion, with gum Arabic, sugar, and mint water. Also, *immediately* after every paroxysm of vomiting, a powder composed of sulph. morph. $\frac{1}{4}$ gr., calomel 1 gr., and acetate of lead 1 gr., mixed with a few grains of sugar and moistened with water, and followed by a piece of ice instead of drink. I mean to be understood literally, when I say *immediately after* every paroxysm of vomiting, for the stomach cannot maintain a continuous effort to eject its contents. Hence, if the powder is swallowed immediately after the contractile power of the stomach has been exhausted by a paroxysm of vomiting, a little time will elapse before another paroxysm can take place, during which the morphia gains some impression on the nerves, and the calomel and lead on the capillaries, of the mucous membrane. Whereas, if you wait, as almost all patients and nurses will desire to do after each act of vomiting, until the patient has "rested a few minutes," or the "stomach has settled a little," you delay exactly long enough for the muscular coat of the stomach to regain its contractility and its cavity to have gathered a fresh accumulation of effused serum, and then if you give the powder, or anything else, in nine trials out of ten, it will be immediately rejected and its effects lost.

At the same time that I direct the foregoing remedies by the mouth, in the exact manner stated, I direct $\frac{1}{2}$ grain of sulphate of morphine and 10 grains of acetate of lead, dissolved in 2 or 3 ounces of cold water, to be injected into the rectum *immediately* after each evacuation from the bowels. When the serous discharges have continued until the water and salts of the blood are so much exhausted as to materially increase the viscidity of that fluid, I then endeavor to replenish the lost materials, and also aid the strychnia in maintaining nerve sensibility, by giving, alternately, every 15 or 20 minutes, a tablespoonful of strong infusion of coffee or tea and animal broth well salted with chloride of sodium or chlorate of potassa.

If the stage of true and full collapse finally ensues, the chances of recovery will be extremely small, but they will be best promoted by withdrawing all remedies but the strychnine, caffein, or nitrous-oxide gas, and the patient, judicious use of animal broth well salted, and dry external warmth.

If, either before or after collapse, febrile reaction comes on, and a low grade of secondary fever is established, it must be treated on the same principles as the lower grades of typhoid or enteric fever. I have thus briefly stated the treatment in which I was induced to place much confidence during an active and somewhat extensive experience in five successive cholera epidemics. I am satisfied that all alcoholic stimulants and what are called *heroic* doses of medicine, of any kind, are injudicious and generally hurtful, in the treatment of this much-dreaded disease.

